# A Low‐Cost Metal‐Free Photocatalyst Based on Black Phosphorus

**DOI:** 10.1002/advs.201801321

**Published:** 2018-11-09

**Authors:** Min Wen, Jiahong Wang, Ruifeng Tong, Danni Liu, Hao Huang, Ying Yu, Zhang‐Kai Zhou, Paul K. Chu, Xue‐Feng Yu

**Affiliations:** ^1^ Center for Biomedical materials and Interfaces Shenzhen Institutes of Advanced Technology Chinese Academy of Sciences Shenzhen 518055 P. R. China; ^2^ Department of Physics and Department of Materials Science and Engineering City University of Hong Kong Hong Kong 999077 P. R. China; ^3^ School of Physics State Key Laboratory of Optoelectronic Materials and Technologies Sun Yat‐sen University Guangzhou 510275 P. R. China

**Keywords:** 2D materials, black phosphorus, hydrogen evolution, photocatalysis

## Abstract

An efficient metal‐free photocatalyst composed of black phosphorus (BP) and graphitic carbon nitride (CN) is prepared on a large scale by ball milling. Using economical urea and red phosphorus (RP) as the raw materials, the estimated materials cost of BP/CN is 0.235 Euro per gram. The BP/CN heterostructure shows efficient charge separation and possesses abundant active sites, giving rise to excellent photocatalytic H_2_ evolution and rhodamine B (RhB) degradation efficiency. Without using a co‐catalyst, the metal‐free BP/CN emits H_2_ consistently at a rate as large as 786 µmol h^−1^ g^−1^ and RhB is decomposed in merely 25 min during visible‐light irradiation. The corresponding electron/hole transfer and catalytic mechanisms are analyzed and described. The efficient metal‐free catalyst is promising in visible‐light photocatalysis and the simple ball‐milling synthetic method can be readily scaled up.

As one of the abundant elements on earth, phosphorus (P) accounts about 0.1%^1^ and one of the allotropes, black phosphorus (BP), has recently attracted a lot of scientific interest as a 2D semiconductor.^2^, ^3^, ^4^, ^5^, ^6^, ^7^, ^8^, ^9^, ^10^, ^11^ Owing to the unique optical and electrical properties, BP holds large promise in the fields of energy and catalysis.^12^, ^13^, ^14^, ^15^, ^16^, ^17^, ^18^, ^19^ In particular, BP has a thickness‐dependent direct bandgap ranging from 0.3 to 2.1 eV, leading to broad absorption spanning the ultraviolet (UV) and near‐infrared (NIR) regions of the solar spectrum.^14^ Furthermore, because of the 2D structure, BP shows a high charge mobility up to 1000 cm^2^ V^−1^ s^−1^ and large surface area.^20^, ^21^ These characteristics are attractive to photocatalysis and the development of heterostructured catalysts. Consequently, various BP‐based heterostructures such as BP/TiO_2_,^22^ BP/CoP,^23^ BP/Au/La_2_Ti_2_O_7_,^24^ BP/BiVO_4_,^25^ and BP/Au/CdS^26^ have been proposed to have good photocatalytic characteristics. However, most of these heterostructures comprise metal semiconductors as the major components and so the associated materials cost tends to be high thus hampering large‐scale industrial adoption. Hence, metal‐free photocatalysts comprising abundant elements and delivering good catalytic performance are highly desirable.

Another metal‐free 2D semiconductor, graphitic carbon nitride (CN), is also attractive to photocatalysis on account of the unique features such as the large surface area, light/thermal stability, and suitable conduction band (CB, −1.0 V vs normal hydrogen electrode (NHE)) and valence band (VB, +1.7 V vs NHE) potential.^27^, ^28^, ^29^, ^30^, ^31^, ^32^ Recently, binary metal‐free heterostructures combining BP and CN have shown promise in photocatalytic H_2_ evolution and reactive oxygen species generation.^33^, ^34^, ^35^, ^36^ Nevertheless, efficient synthesis of BP/CN heterostructures is still challenging and the BP materials used in production are typically limited to ultrathin nanosheets and quantum dots. The preparation of these materials relies on the growth and exfoliation of BP crystals and the production cost and complexity increase inevitably. All in all, an economical synthetic technique to prepare BP‐based heterostructured photocatalysts is still lacking.

Herein, we describe a new BP/CN heterostructured photocatalyst comprising BP nanoparticles with a CB minimum (CBM) potential of ≈−0.31 V (vs NHE)^12^ suitable for proton reduction and reactive oxygen species generation. The BP/CN heterostructure is synthesized by a simple ball‐milling technique which yields several grams of products each time. By using economical urea and red phosphorus (RP) as the raw materials, the materials cost of BP/CN is 0.235 Euro per gram. The metal‐free heterostructure shows broad light absorption spanning the UV to NIR regions, enhanced charge separation efficiency, abundant active sites, featuring excellent photocatalytic activity. During visible‐light irradiation, the BP/CN heterostructure not only emits H_2_ consistently at a rate of 786 µmol h^−1^ g^−1^ but also decomposes rhodamine B (RhB) in only 25 min. The photocatalytic efficiency is comparable to that of previously reported BP/CN heterostructures composed of BP nanosheets or quantum dots.^19^, ^33^, ^35^, ^36^ The efficient BP/CN heterostructured catalyst and low‐cost synthetic process suggest large potential in visible‐light photocatalytic applications.

The preparation procedures and structure of the BP/CN heterostructures are presented in **Scheme** **1**. The BP powder is synthesized from RP by high‐energy ball milling at 1500 rpm for 7 h. The CN powder is prepared from urea at 550 °C in air for 2 h.^37^ BP and CN are mixed in ethanol and after ball milling at 200 rpm for 5 h, the BP/CN heterostructure is produced (yield of several grams each time). By changing the amount of additives, BP/CN heterostructures with different BP mass ratios are prepared and the samples are designated as 1% BP/CN, 5% BP/CN, 10% BP/CN, and 20% BP/CN. For 10% BP/CN, the materials cost is calculated to be 0.235 Euro in which BP accounts for 0.002 Euro (see detailed calculation in Supporting Information). In addition, ball milling is a simple and common industrial technique boding well for large‐scale production of the BP/CN heterostructures.

**Scheme 1:**
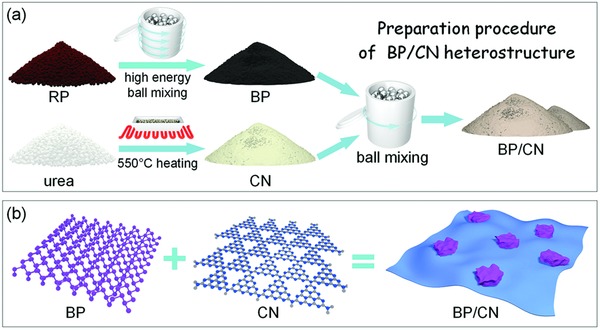
a) Preparation procedures and b) structures of the BP, CN, and BP/CN heterostructure.

The bare BP and CN samples are characterized by X‐ray diffraction (XRD) and Raman scattering to verify successful preparation (Figures S1 and S2, Supporting Information). To clarify the construction of the BP/CN heterostructures, XRD patterns are acquired from the BP, CN, and BP/CN samples with different BP contents. As shown in **Figure** **1**a, the heterostructures show characteristic diffraction peaks of BP and CN but no impurity peaks indicating that they are two‐phase composites. As the mass ratio of BP increases, the peaks of CN gradually decline and those of BP become more intense. The Raman spectra in Figure S3 in the Supporting Information confirm the XRD results. The emerging peaks at 358.4, 430.6, and 460.1 cm^−1^ are attributed to the *A* ^1^ *_g_*, *B*_2_ *_g_*, and *A* ^2^ *_g_* phonon modes of BP, demonstrating the construction of the heterostructures. The typical morphology of BP, CN, and 10% BP/CN heterostructures is examined by scanning electron microscopy (SEM) and transmission electron microscopy (TEM). As shown in Figure 1b and Figure S4 in the Supporting Information, most BP nanoparticles have a size of 50–150 nm. From Figure S5 in the Supporting Information, the CN samples are porous nanosheets with rough surfaces and rolling edges. In the BP/CN heterostructure, the BP nanoparticles are randomly embedded in CN (Figure 1c) and the C, N, and P elemental maps confirm the random distribution (Figure S6, Supporting Information).

**Figure 1:**
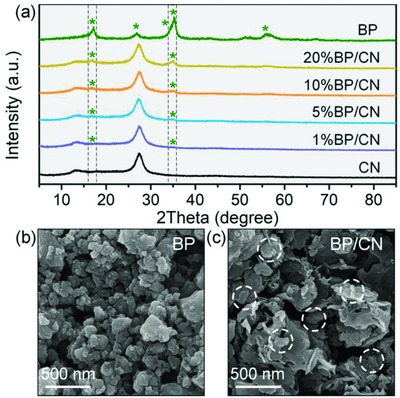
a) XRD patterns of BP, CN, and BP/CN. SEM images of b) BP and c) 10% BP/CN.

The typical single‐sheet TEM image of the BP/CN heterostructure is shown in **Figure** **2**a in which a BP nanoparticle is attached to the CN nanosheets. The high‐angle circular dark field (HAADF) image of BP/CN and corresponding energy dispersive spectroscopy (EDS) maps of C, N, and P verify the composition of the heterostructure (Figure 2b). The high‐resolution TEM (HR‐TEM) image is displayed in Figure 2c. Similar to previous reports, no obvious lattice fringe can be observed from the CN nanosheets.^33^, ^35^ The apparent lattice space of 0.262 nm can be assigned to BP nanoparticle and corresponds to the (040) plane. The results demonstrate that BP nanoparticles and CN nanosheets are integrated into the heterostructure by simple ball milling.

**Figure 2:**
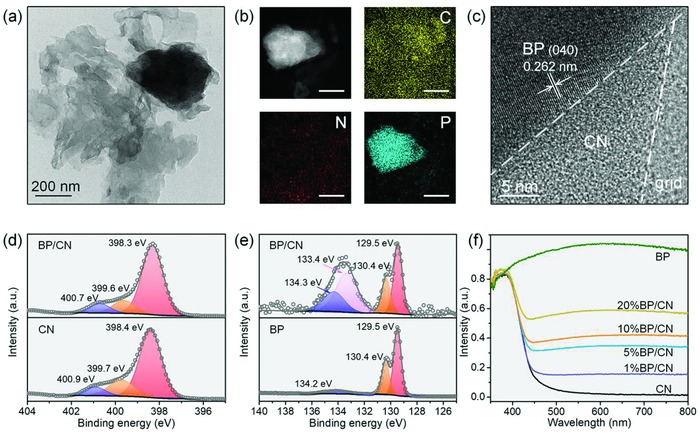
a) TEM image of BP/CN. b) HAADF image of BP/CN and elemental maps of C, N, and P. c) HR‐TEM image of BP/CN. d) High‐resolution N 1s spectra of CN and BP/CN. e) High‐resolution P 2p spectra of BP and BP/CN. f) Absorption spectra of BP, CN, and BP/CN.

The chemical states of BP/CN and interfacial interactions are investigated by X‐ray photoelectron spectroscopy (XPS). The XPS spectrum in Figure S7 in the Supporting Information confirms the existence of C, N, and P in the BP/CN heterostructure. The high‐resolution C 1s spectrum of BP/CN shows two peaks corresponding to sp^2^ C—C bonds (284.5 eV) and sp^2^‐hybridized carbon in the aromatic N—C=N (287.8 eV), respectively (Figure S8, Supporting Information).^38^, ^39^ The high‐resolution N 1s spectrum is composed of three peaks with energies of 398.3, 399.6, and 400.7 eV corresponding to sp^2^ bonded N (C—N=C) in the triazine rings, tertiary nitrogen groups (N—C_3_) in the skeleton, and surface uncondensed C—N—H amino groups, respectively (Figure 2d).^40^ The XPS spectrum of P 2p of BP/CN shows the BP binding energies at 129.5 and 130.4 eV attributed to 2p_3/2_ and 2p_1/2_, respectively (Figure 2e). Besides the typical peaks of BP and phosphorus oxide (134.3 eV), a new peak is found at 133.4 eV and can be assigned to P—N coordination which indicates the strong interaction between BP and CN and confirms their integration.^41^, ^42^, ^43^, ^44^ The chemical interaction between BP and CN is further verified by the solid‐state ^31^P NMR. As shown in Figure S9 in the Supporting Information, besides the characteristic peak of P—P at 17.6 ppm, a new peak at 5.5 ppm is observed, confirming the formation of P—N coordinate bonds in BP/CN.^33^

The optical absorption properties of the BP, CN, and BP/CN heterostructures are determined from the UV–vis–NIR diffuse reflectance spectra. As shown in Figure 2f, pristine CN with a bandgap of about 2.7 eV shows an absorption edge at 450 nm, whereas bare BP (0.3 eV) exhibits broad absorption from 350 to 800 nm. The BP/CN heterostructure shows broad absorption in the UV and NIR regions and stronger visible‐light absorption can be achieved if the BP ratio is increased. The optical changes are also illustrated by the color transition of the BP/CN samples. When the BP content goes up, the color of the heterostructure changes from milky white to dark yellow and then to dark gray (Figure S10, Supporting Information).

The photocatalytic properties of the BP/CN heterostructure are first investigated based on H_2_ evolution during visible‐light irradiation. Without co‐catalysts (metal complex, noble metal, etc.), BP/CN is dispersed directly in water containing isopropanol (IPA) for photocatalytic H_2_ generation. The mixture is illuminated by a blue LED lamp (440–445 nm) and the amount of H_2_ emitted is determined by gas chromatography using methane as the internal standard. As shown in **Figure** **3**a, a trace amount of H_2_ is detected when bare BP or CN serves as the photocatalyst. In contrast, the BP/CN heterostructure produces H_2_ continuously, demonstrating their efficiency for photocatalytic H_2_ evolution. The H_2_ evolution rate goes up as the BP ratio is increased from 1 to 10 wt%. However, when the BP ratio is further increased to 20 wt%, the relative decrease of CN reduces the photocatalytic efficiency. The optimal BP/CN structure produces H_2_ evolution at rate of 786 µmol h^−1^ g^−1^, which is comparable to those of previously reported BP/CN heterostructures consisting of BP nanosheets and quantum dots (Figure 3a and Table S1, Supporting Information).

**Figure 3:**
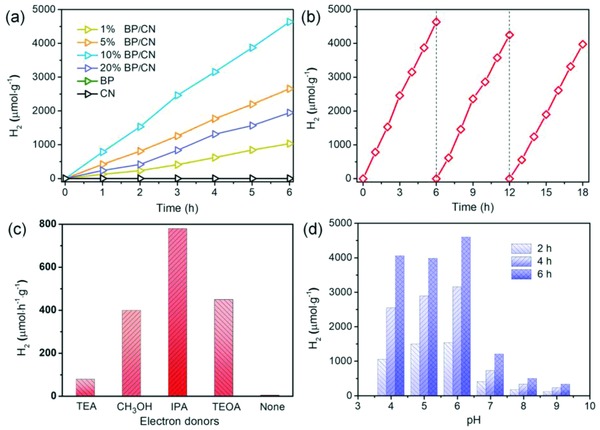
a) Photocatalytic H_2_ evolution from BP, CN, and BP/CN in water containing IPA. b) Cycle stability of 10% BP/CN for photocatalytic H_2_ evolution. c) Photocatalytic H_2_ evolution from 10% BP/CN with different electron donors. d) Photocatalytic H_2_ evolution from 10% BP/CN for different pH values.

To evaluate the stability of the photocatalyst, the optimal 10% BP/CN is recycled three times (Figure 3b) and continuous H_2_ evolution with no obvious degradation is observed. The slight decay can be attributed to the loss of BP/CN during centrifugation and recollection. Apart from IPA, BP/CN can catalyze H_2_ evolution effectively when combined with different electron donors such as methanol (CH_3_OH), triethanolamine (TEOA), and trimethylamine (TEA) (Figure 3c). Hence, the metal‐free BP/CN is efficient in producing H_2_ evolution in different systems. Photocatalytic experiments performed at different pH values show that BP/CN can emit H_2_ over a broad pH range (Figure 3d). Photocatalytic experiments performed at different pH values show that BP/CN can emit H_2_ over a broad pH range (Figure 3d). Obviously, the H_2_ evolution efficiency under acidic condition is much better than that in alkaline solutions. This pH‐dependent effect is related to the protonation of BP/CN and the chemical state of IPA. On one hand, the protonated BP/CN can accelerate the trap of electrons and promote the H_2_ evolution.^33^ On the other hand, the increase of protons will change the chemical states of IPA (p*K*_a_ = 15.6), affecting the efficiency as a result.^45^, ^46^, ^47^ In consideration of the synergetic influences, the optimized pH is found to be 6.0 in this system.

The efficiency of charge separation plays an important role in photocatalysis. Steady‐state and time‐resolved photoluminescence spectra are thus acquired to investigate the charge separation efficiency of pristine CN and BP/CN. As shown in **Figure** **4**a, a broad emission peak at 455 nm is observed. In comparison with pristine CN, the emission peak from 10% BP/CN blue‐shifts slightly and the emission intensity is quenched by ≈50%. The results suggest that the recombination of photogenerated electron/hole pairs in BP/CN is mitigated. In the time‐resolved photoluminescence spectra in Figure 4b and Table S2 in the Supporting Information, the average lifetime of BP/CN is found to be 1.93 ns that is longer than that of bare CN (0.96 ns). The longer photoluminescence lifetime suggests that the BP combination can prolong charge carrier separation in CN.^35^, ^48^ Therefore, in comparison with bare CN, the photogenerated carriers in BP/CN heterostructure are more likely to participate in the subsequent chemical reactions.

**Figure 4:**
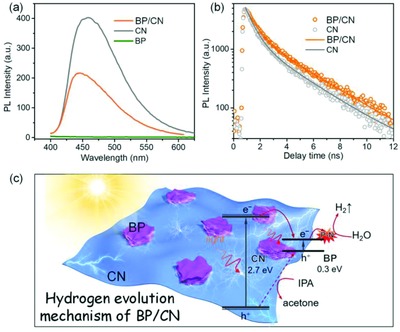
a) Photoluminescence spectra and b) time‐resolved photoluminescence spectra of CN and BP/CN. c) Schematic of the charge transfer process and mechanism of photocatalytic H_2_ emission.

To further examine the improved charge separation efficiency of BP/CN in the photocatalytic H_2_ evolution system, the photoelectrochemical water splitting characteristics are studied. As shown in Figure S11 in the Supporting Information, the photocurrent density of BP/CN is higher than that of CN, indicating facilitated charge separation in the BP/CN heterostructure. Electrochemical impedance spectroscopy is performed to confirm the charge transfer properties of BP, CN, and BP/CN samples. According to the Nyquist plots and the corresponding equivalent circuit model in Figure S12 in the Supporting Information, BP/CN features a smaller interfacial charge transfer resistance (R_CT_, Table S3, Supporting Information). In comparison with bare BP and CN, the smaller R_CT_ of BP/CN samples suggests a decreased charge transfer resistance and an accelerated charge migration.

The possible charge transfer processes in the photocatalytic H_2_ evolution system are described in Figure 4c. According to the corresponding CB and VB potentials, BP/CN is a type‐I heterostructure. When BP/CN is exposed to visible light, CN and BP are excited to generate electron/hole pairs. The photogenerated electrons in the CB (−1.0 V vs NHE) of CN are aligned with the exothermal transfer to BP (−0.31 V vs NHE). Therefore, the adjacent BP acts as the electron acceptor to inhibit charge recombination of the photogenerated carriers and the electrons on the CB of BP are trapped by the interfacial P—N bond for the subsequent H_2_ evolution.^33^ On the other hand, the photogenerated holes in CN (1.7 V vs NHE) are more likely to be eliminated by IPA directly since the VB of BP (−0.01 V vs NHE) is more negative than the oxidation potential of IPA (0.12 V vs NHE).^49^ Based on the above discussion, H_2_ generation of BP/CN stems from promoted charge separation and efficient catalytic sites.

RhB degradation experiments are also performed to investigate the visible‐light photocatalytic performance of the BP/CN heterostructure. As shown in Figure S13 in the Supporting Information, the absorption peak of RhB at 554 nm decreases gradually as the irradiation time prolongs. **Figure** **5**a and Figure S14 in the Supporting Information show the amounts of RhB under different conditions demonstrating that BP/CN delivers better performance than the bare CN and BP. The optimal photocatalyst is 5% BP/CN which can decompose RhB in merely 25 min. A larger BP ratio does not always produce better performance because the CN content decreases correspondingly. Further experiments demonstrate that the BP/CN can degrade RhB at different temperatures (Figure S15, Supporting Information). Even at a low temperature of 5 °C, the RhB can also be degraded in 25 min. The photocatalytic stability is further assessed by repeating the degradation experiments several times. As shown in Figure S16 in the Supporting Information, the photocatalytic performance is similar in the three trials indicating that BP/CN has good stability. The good efficiency and stability are mainly benefit from the interactions between BP and CN since the physical mixture of BP and CN (BP + CN) delivers poorer performance (Figure S17, Supporting Information).

**Figure 5:**
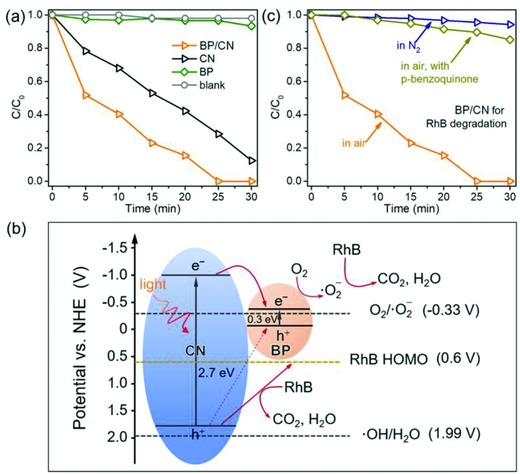
a) Photocatalytic characteristics of BP, CN, and BP/CN in RhB degradation in water under visible‐light irradiation. b) Schematic illustration of the VB and CB of BP/CN and photocatalytic mechanism for RhB degradation. c) Photocatalytic performance of BP/CN under different conditions.

The photocatalytic mechanism of BP/CN in RhB degradation is investigated and Figure 5b shows the potentials and possible charge transfer process. Thermodynamically, the photogenerated electrons are able to reduce molecular O_2_ to·O_2_^−^ since the CBM potential of BP is similar to the redox potential of O_2_/·O_2_^−^. However, the photogenerated holes cannot oxidize H_2_O to ·OH and therefore, the generated ·O_2_^−^ seems to be the protagonist against RhB degradation. Control experiments are designed to verify this hypothesis. As shown in Figure 5c, photodegradation of RhB in N_2_ is very weak meaning that O_2_ is essential in the current photocatalysis. To further confirm the effects of ·O_2_^−^, *p*‐benzoquinone is introduced to the solution. As an effective scavenger for ·O_2_^−^, *p*‐benzoquinone consumes ·O_2_^−^ in the solution and as a result, the degradation efficiency decreases significantly from 100% to 11% after addition of *p*‐benzoquinone, corroborating that ·O_2_^−^ is the reactive oxygen species responsible for photocatalysis. Considering the good stability of the catalyst, the photogenerated holes on the VB of CN are captured timely. Since the highest occupied molecular orbital (HOMO) potential of RhB (0.6 V vs NHE, Figure S18, Supporting Information) is more negative, holes can be consumed by RhB for oxidation.^35^, ^50^ Based on above discussion, the degradation of RhB stems from the photogenerated ·O_2_^−^ and holes. Unlike the photocatalytic H_2_ evolution, the degradation reaction does not depend on the active sites of P—N. Beyond the optimal ratio of 5%, the relative decrease of CN will result in a decreased ·O_2_^−^ and holes. So 5% BP/CN exhibits the superior efficiency for RhB degradation while 10% BP/CN shows better performance in H_2_ evolution.

In conclusion, a metal‐free BP/CN heterostructured photocatalyst is prepared from economical RP and urea by simple ball milling. The calculated materials cost is 0.235 Euro per gram due to the cheap raw materials. This heterostructure shows excellent and stable visible‐light photocatalytic properties in not only H_2_ evolution (786 µmol h^−1^ g^−1^) but also rapid (25 min) degradation of RhB. The excellent photocatalytic activity and stability are attributed to efficient charge separation and migration in the type‐I band alignment, as well as formation of P—N bonds in the BP/CN heterostructure. Since the synthesis is simple, economical, and scalable, the efficient metal‐free catalyst has large potential in visible‐light photocatalysis and the ball milling technique can serve as a general technique to produce heterostructures on a large scale.

## Conflict of Interest

The authors declare no conflict of interest.

## Supporting information

Supplementary
