# Supplementary material for: A Low‐Cost Metal‐Free Photocatalyst Based on Black Phosphorus
Source: Adv Sci (Weinh). 2018 Nov 9;6(1):1801321. doi: 10.1002/advs.201801321 (PMC6325597; doi:10.1002/advs.201801321)
Supplement: Supplementary file 1 — Supplementary [file ADVS-6-1801321-s001.pdf]

Copyright WILEY-VCH Verlag GmbH & Co. KGaA, 69469 Weinheim, Germany, 2018.

ADVANCED SCIENCE
Open Access

Supporting Information

for *Adv. Sci.,* DOI: 10.1002/advs.201801321

A Low-Cost Metal-Free Photocatalyst Based on Black Phosphorus

*Min Wen, Jiahong Wang, Ruifeng Tong, Danni Liu, Hao Huang, Ying Yu, Zhang-Kai Zhou, Paul K. Chu, and Xue-Feng Yu**

Copyright WILEY-VCH Verlag GmbH & Co. KGaA, 69469 Weinheim, Germany, 2016.

Supporting Information

A Low-Cost Metal-Free Photocatalyst Based on Black Phosphorus

Min Wen, Jiahong Wang, Ruifeng Tong, Danni Liu, Hao Huang, Ying Yu, Zhang-Kai Zhou, Paul K. Chu, Xue-Feng Yu*

Experimental Section

1. Chemicals

Urea, red phosphorus (RP), rhodamine B (RhB), ethanol, and p-benzoquinone were purchased from Aladdin and P25 was obtained from Degussa AG. Ultrapure water with a resistivity of 18.2 MΩ·cm was used in the experiments and all the chemicals were used without further purification.

2. Methods

(1) Synthesis of carbon nitride (CN)

The CN nanosheets were synthesized by a reported thermal polymerization method with slight modification.[1] Specifically, urea (10 g) was put into a covered crucible and heated in an muffle furnace in air at a heating rate of 2.3 °C/min to 550 °C. Light-yellow CN was obtained by heating at 550 °C for 2 h and the productivity was 6%.

(2) Synthesis of black phosphorus (BP)

The RP powder (2 g) and stainless steel balls were sealed in a steel vessel in a glove box under Ar. The vessel was then put in the high energy ball-milling machine (Retsch, Emax) and transformation from RP to BP nanoparticles was carried out by ball milling for 7 h at 1500 rpm. The productivity was 100%.

(3) Synthesis of BP/CN heterostructures

The BP/CN heterostructures were synthesized by a wet ball milling method. Briefly, 1.6

g CN and a specific amount of BP were dispersed in 80 ml of ethanol by sonication for 5 minutes. The suspension was sealed in a polytetrafluoroethylene vessel and an air-cooled planetary ball-milling machine (MITR-YXQM-2L) was employed to synthesize the BP/CN heterostructures. After ball milling for 5 h at 200 rpm, the product was collected by centrifugation, washed with ethanol, and dried in a vacuum oven overnight.

(4) Caculation of material cost

Urea (500 g) and RP (500 g) were purchased from Aladdin for 7.75 and 11.1 Euro, respectively.[2-3] The productivity of CN and BP was 6% and 100%, respectively. For 10% BP/CN, the materials cost was calculated to be 0.235 Euro per gram, of which CN accounted for 0.233 Euro and BP 0.002 Euro.

(5) Photocatalytic experiments on RhB degradation

In photocatalytic degradation of RhB, 2.0 mg of the photocatalyst were suspended in 8 mL of the RhB aqueous solution (5.0 $mg\cdot L^{-1}$), equivalent to a chemical oxygen demand (COD) number of 3.3 $mg\cdot L^{-1}$. Before irradiation, the suspension was stirred in darkness for 0.5 h to achieve the adsorption-desorption equilibrium of RhB before exposure to a LED lamp (440-445 nm). The temperature of the photocatalytic system was kept at room tempreature with flowing water. The amount of RhB was determined by UV-Vis spectrophotometry (Hitachi U-3900) and Lambert-Bill Law. The photocatalytic performance of the samples was calculated based on the the decreased amount of RhB. The $\cdot O_2^-$ capture experiment was carried out by addition of p-benzoquinone (0.1 mmol) while other conditions were the same.

(6) Photocatalytic experiments for H_2 evolution

The BP/CN (2.0 mg) was dispersed in a mixture of ultrapure water and isopropanol (10 mL) in a Schlenk tube. The sample was saturated with argon and 500 μL of CH_4 were injected as the internal standard for quantitative GC-TCD analysis. The light source was a blue LED lamp (λ = 440-445 nm) equipped with an agitator and a cooling apparatus. After

irradiation for a certain period of time, 200 μL of the mixed gas were taken from the sample tube and subjected to GC analysis. In the photocatalytic experiments for different pH values, the pH of the ultrapure water and isopropanol solution was adjusted with NaOH or HCl.

Table S1. Reported photocatalytic H_2 evolution efficiency of BP/CN.

Photocatalyst	The used BP	Rate (μmol g^{-1} h^{-1})	Light source
20% BP/CN	ultrathin nanosheets	427	Xe (>420 nm)[5]
1.8% BP/CN	few-layer nanosheets	571	Xe (>400 nm)[6]
7% BP/CN	BP quantum dots	900	LED (420 nm)[7]
5% BP/CN/Pt	BP quantum dots	271	Xe (>420 nm)[8]

(7) Electrochemical cyclic voltammetry

The potential of RhB was determined on the CHI 760E electrochemical workstation. Cyclic voltammetry was performed using a three-electrode configuration in which the glassy carbon electrode with the sample served as the working electrode, saturated calomel electrode (SCE) electrode as the reference electrode, platinum wire as the counter electrode, and Na_2SO_4 (0.1 M) as the electrolyte. The scanning rate was 50 $mV \cdot s^{-1}$.

(8) Photoelectrochemical experiments

The photoelectrochemical measurements were carried out using a typical three-electrode setup in which the ITO glass pasted with the sample was the working electrode, saturated calomel electrode (SCE) was the reference electrode, and platinum sheet was the counter electrode. The Na_2SO_4 (0.1 M) was the electrolyte and the photocurrents were monitored under a bias of -0.3 V (vs NHE).

(9) Calculation of the *average fluorescence lifetime*

The fluorescence decay of the CN and BP/CN heterostructure was studied. The decay traces were fitted with the double-exponential function Y(t) based on nonlinear least-square using the following expression:[4]

$$\tau = \sum_{i=1}^{n} B_i \tau_i^2 / \sum_{i=1}^{n} B_i \tau_i$$

where, B_i and τ_i represent the individual pre-exponential and lifetime terms, respectively, and τ was the average lifetime.

Table S2. The fitted fluorescence decay components of CN, BP/CN

samples	τ_1 (ns)	B_1	τ_2 (ns)	B_2	τ (ns)
BP/CN	0.75	1.33	3.12	0.32	1.93
CN	0.63	1.58	2.88	0.34	0.96

3. Characterization

X-ray diffraction (XRD) was performed on the Rigaku MiniFlex 600 X-ray diffractometer (Cu K_α radiation, 0.15418 nm) and the Raman scattering spectra were acquired at room temperature on a Horiba Jobin-Yvon LabRam HR high-resolution confocal Raman microscope equipped with a 633 nm laser as the excitation source. Scanning electron microscopy (SEM) was conducted on the Zeiss Supra 55 high-resolution field-emission scanning electron microscope at 5.0 kV accelerating voltage and the element maps were obtained at 15.0 kV. The transmission electron microscopy (TEM) and high-angle annular dark-field (HAADF) images were obtained on a field-emission transmission electron microscope (JEOL JEM 3200Fs) at 200 kV. The single sheet element maps were acquired on the JEOL JEM 3200Fs at 300 kV equipped energy dispersive X-ray spectroscopy (EDS). X-ray photoelectron spectroscopy (XPS) was conducted on the Thermo Fisher Escalab 250Xi using monochromatic Al K_α radiation. The UV-Vis-NIR diffuse reflectance spectra (DRS) were acquired on the Shimazu UV-2450 spectrophotometer and the steady-state

photoluminescence (PL) spectra were obtained on a fluorescence spectroscopy (PerkinElmer LS55). The time-resolved fluorescence spectra were taken using a mode lock Ti:Sapphire laser (MaiTai, Spectra Physics) with a pulse width of ~100 fs and repetition rate of 79 MHz. Luminescence from the sample was monitored by a liquid-nitrogen-cooled CCD (SPEC-10, Princeton) and the decay traces (photoluminescence) were recorded using a time-correlated single-photon counting system (PicoQuant GmbH).

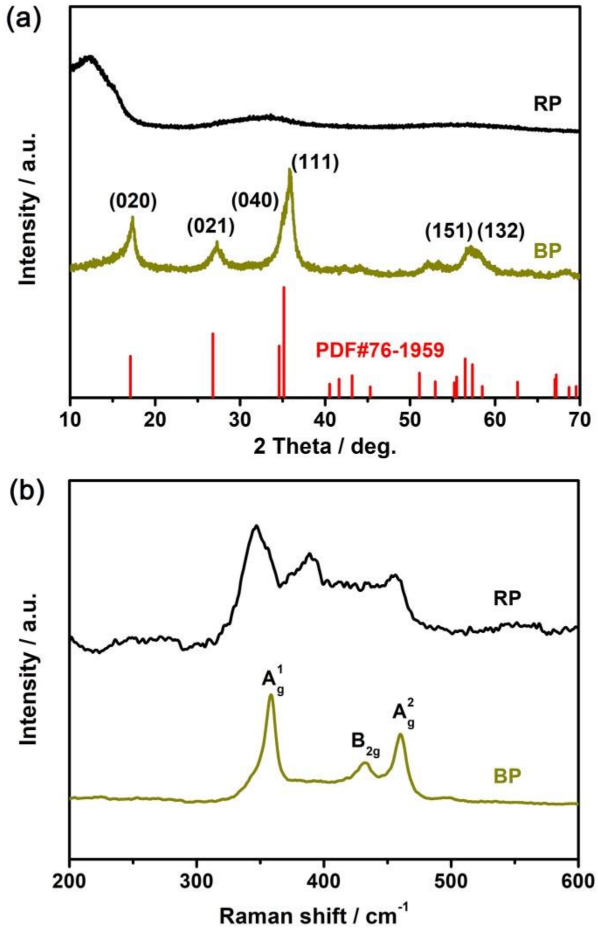

Figure S1. (a) XRD patterns and (b) Raman spectra of RP and BP.

As shown in Figure S1a, the XRD pattern of BP nanoparticles exhibit distinct peaks at 17.3° (020), 26.9° (021), 34.5° (040), and 35.3° (111) (PDF#79-1959), which are quite different from the weak broad peaks from amorphous RP.[9] The Raman scattering spectra show three prominent peaks at 358.4, 431.7, and 460.1 cm^{-1} attributed to the A^1_g, B_{2g}, and A^2_g phonon modes of BP, respectively (Figure S1b). These results confirm transformation of BP

from RP.

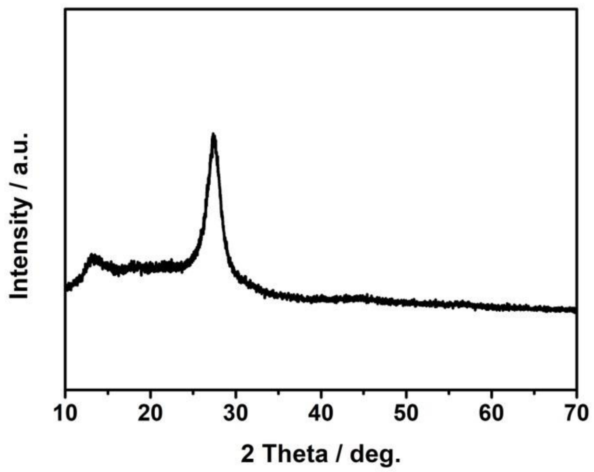

Figure S2. XRD pattern of CN. The characteristic peaks at 13.1° (100) and 27.3° (002) verify synthesis of CN.

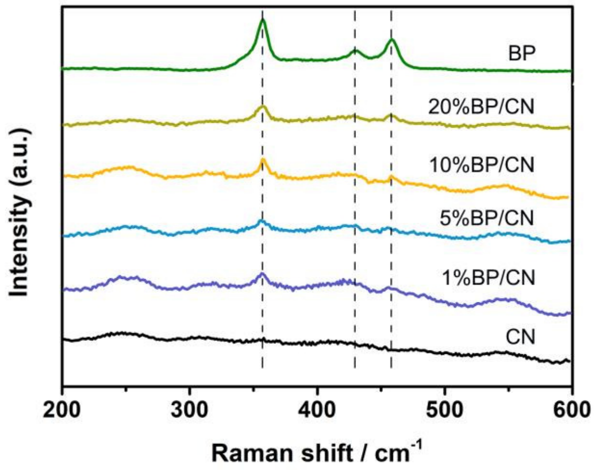

Figure S3. Raman spectra of BP, CN and BP/CN samples.

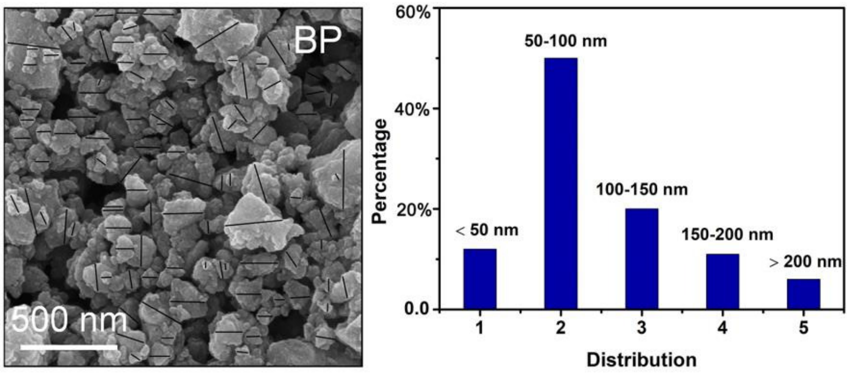

Figure S4. SEM image and size distribution of BP nanoparticles.

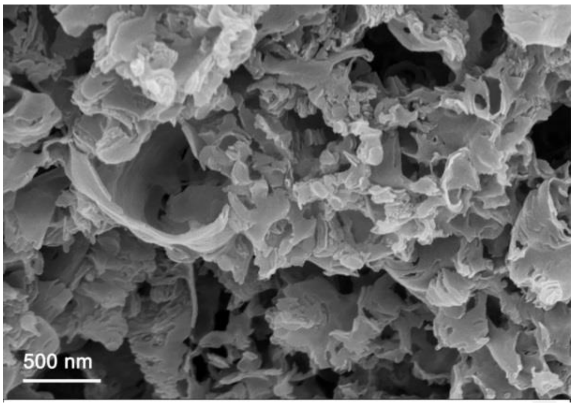

Figure S5. SEM image of CN.

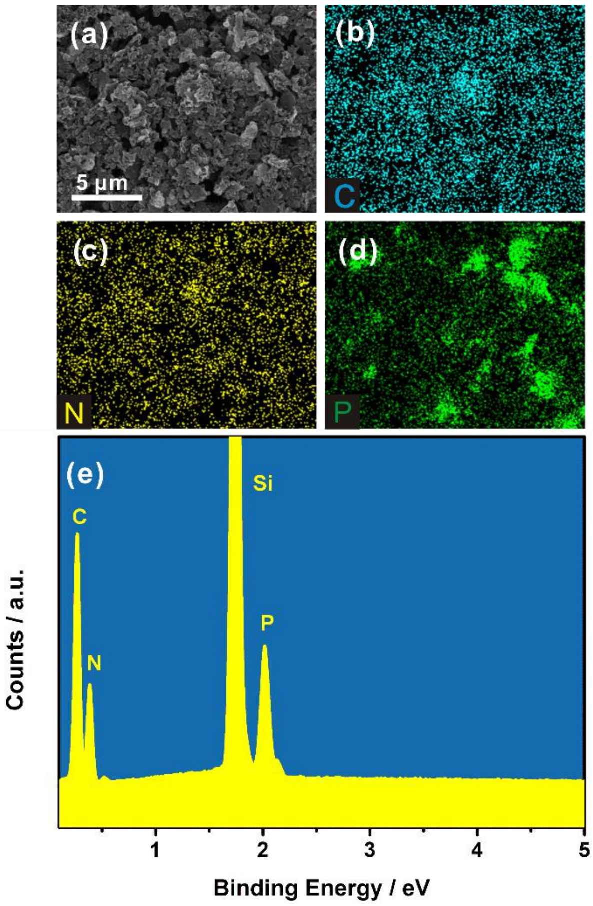

Figure S6. (a) SEM image of 10% BP/CN and corresponding elemental maps of (b) C, (c) N, and (d) P; (e) EDS spectrum of 10% BP/CN.

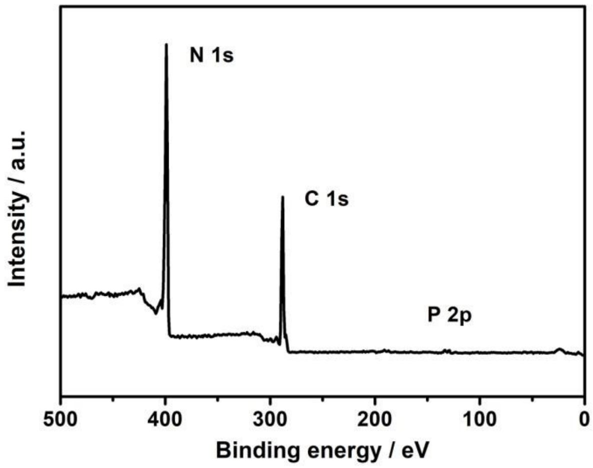

Figure S7. XPS spectrum of BP/CN.

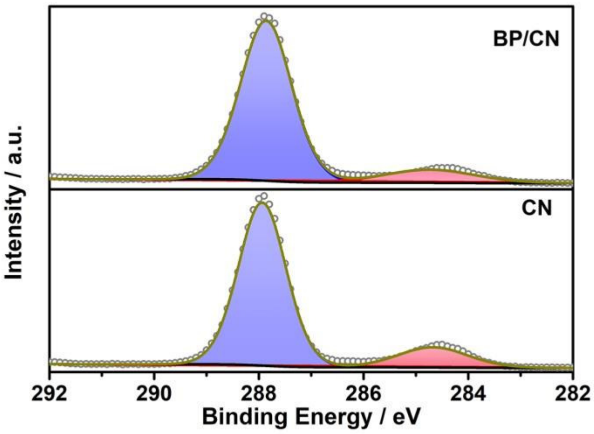

Figure S8. High-resolution C (1s) spectra of CN and BP/CN.

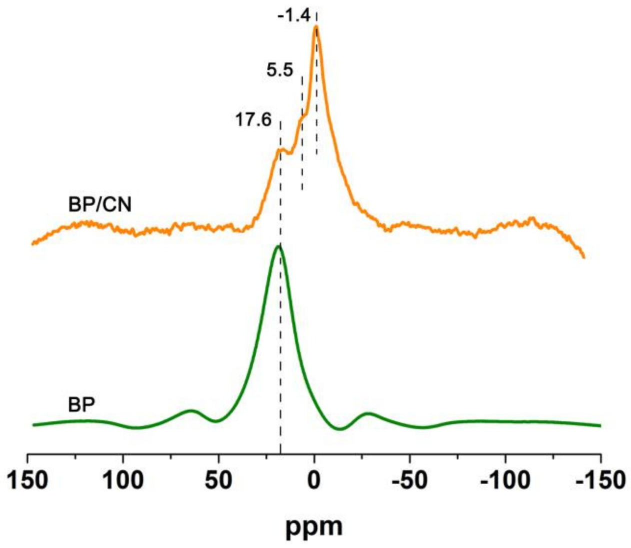

Figure S9. Solid–state ^{31}P NMR spectra of BP/CN and BP.

The peaks at 17.6, 5.5, and -1.4 ppm are attributed to the P-P bonds of BP, the P-N coordinate bonds and the oxidized phosphorus in BP/CN.

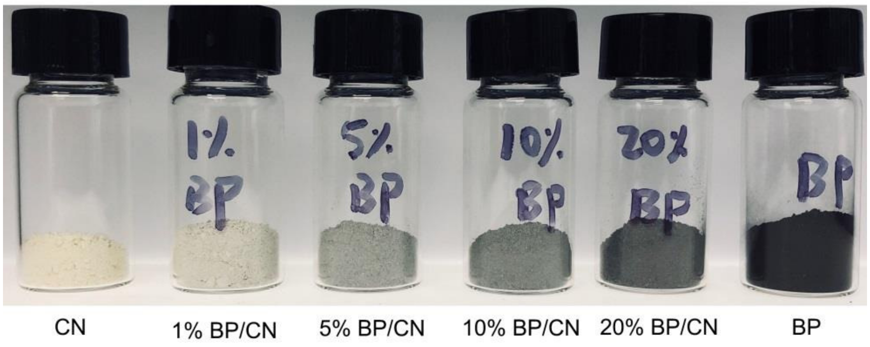

Figure S10. Photographs of the CN, BP, and BP/CN samples.

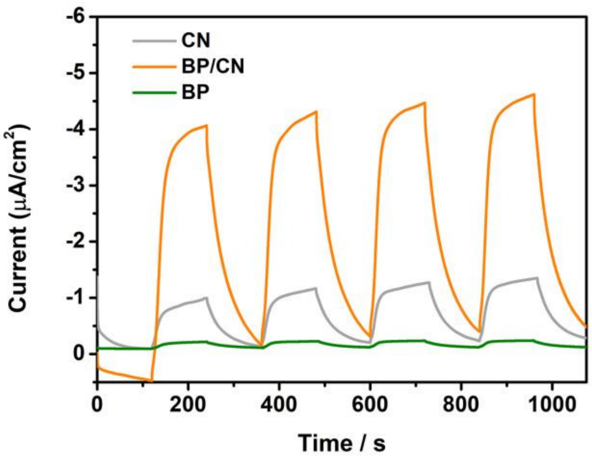

Figure S11. Photocurrent response of BP, CN and 10% BP/CN at a bias of -0.3 V (vs NHE).

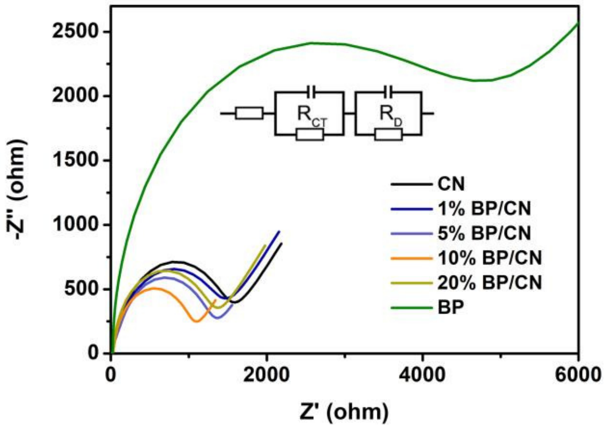

Figure S12. EIS Nyquist plots and the equivalent circuit (inset) of BP, CN and BP/CN samples measured at 0.7 V (vs NHE) under visible-light irradiation with Na_2SO_4 (0.1 M) as the electrolyte.

The electrical equivalent-circuit model (inset) is used to simulate the EIS test, where R_{CT} is the charge transfer resistance at the electrode/electrolyte interface, R_D is the diffusion resistance. The relative variation of BP, CN and BP/CN samples is in accordance with the reported work, where BP/CN samples feature smaller charge transfer resistance.[4-5] The calculated values of the equivalent circuit elements are shown in Table S3.

Table S3. Calculated values of equivalent circuit elements for the samples.

Samples	CN	1% BP/CN	5% BP/CN	10% BP/CN	20% BP/CN	BP
R_{CT} (Ω)	1619	1518	1274	1087	1439	5588

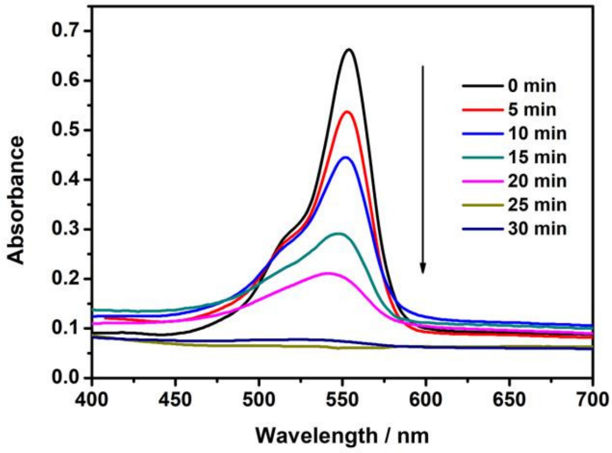

Figure S13. UV-vis absorption spectra of RhB for different irradiation time for 5% BP/CN.

The photocatalytic experiments are carried out by dispersing the photocatalysts in the RhB aqueous solution (5.0 mg·L^{-1}) and irradiation with a bule LED lamp (440-445 nm). The pollutant degradation rates are determined by characteristic absorption of RhB, which decreases gradually with exposure time.

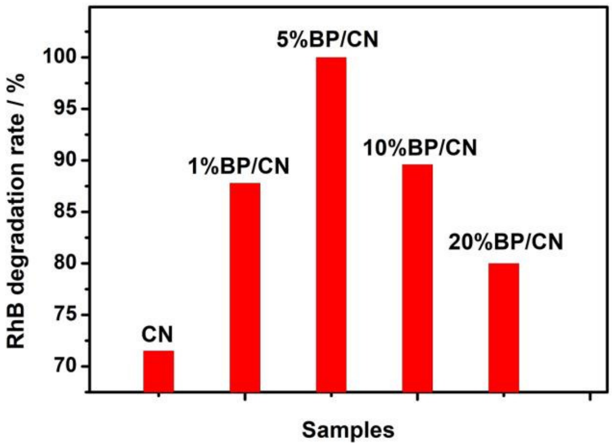

Figure S14. Effects of BP mass ratio in BP/CN on RhB degradation.

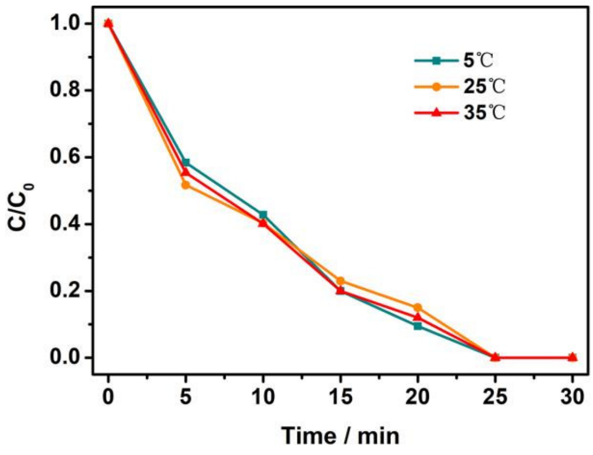

Figure S15. Photocatalytic efficiency of 5% BP/CN on RhB degradation under different temperatures.

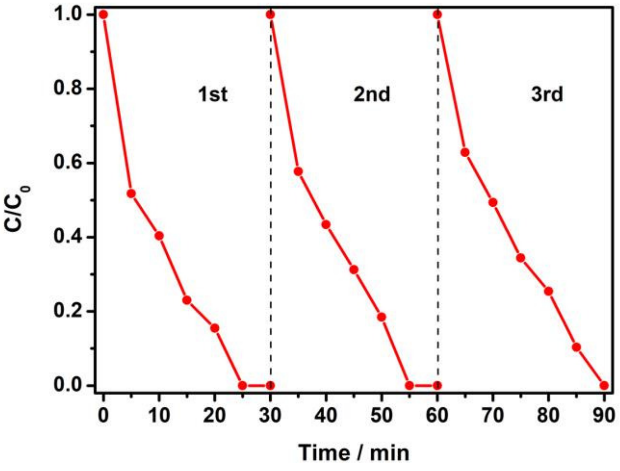

Figure S16. Cycling stability of 5% BP/CN in the RhB degradation experiment.

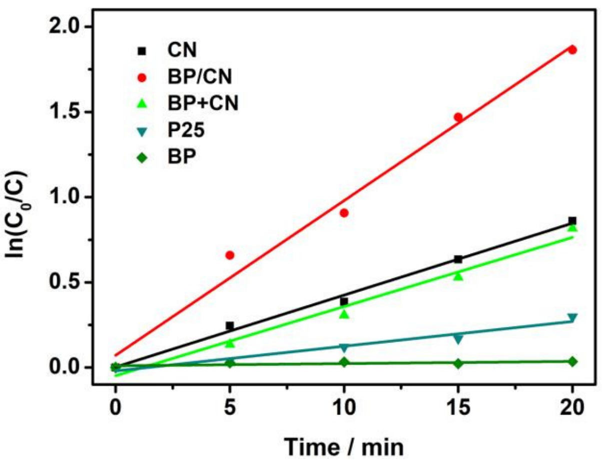

Figure S17. Comparison of degradation rates among BP, CN, 5% BP/CN, BP+CN mixture, and P25.

To verify the effects of the interaction between CN and BP on photocatalysis, a control photodegradation experiment is performed with the physical mixture of BP and CN (BP+CN). For the same components ratio, the physical mixture of BP and CN shows lower photocatalytic efficiency than the BP/CN heterostructure and CN.

According to the references,[4,10] the degradation of RhB undergoes the following procedures: 1) N-de-ethylation, 2) Chromophore cleavage, 3) Opening-ring, 4) Mineralization and the final product is CO_2 and H_2O.

The concentration of RhB and the time fits the following equation:

$$\ln C_0/C = kt$$

where C_0 is the original concentration of RhB, C is the concentration of RhB at different times. The characteristic equation demonstrates that the degradation of RhB is a first order reaction. The degradation rate constant of 5% BP/CN is up to 0.10 min^{-1}, which is about 2.6 times that of the CN and BP mixture (0.038 min^{-1}), 2.3 times that of bare CN, 9 times that of commercial P25 (0.011 min^{-1}), and 77 times that of bare BP (0.0013 min^{-1}).

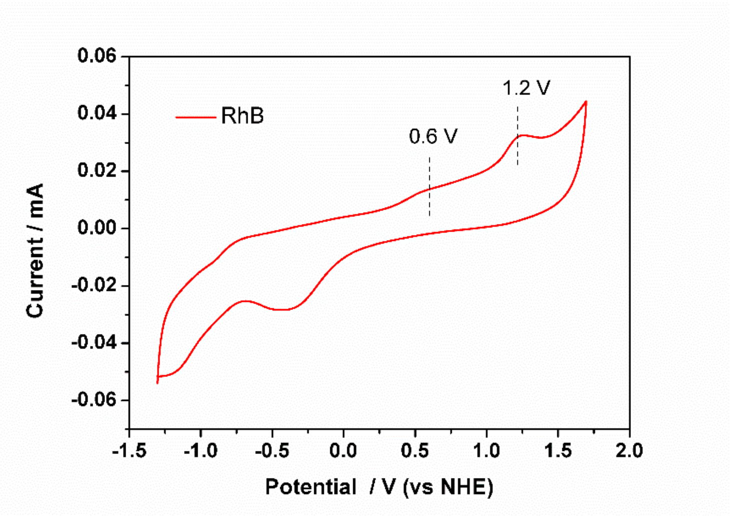

Figure S18. CV curve of RhB.

References

[1] J. Liu, T. Zhang, Z. Wang, G. Dawson, W. Chen, J. Mater. Chem. **2011**, 21, 14398.

[2] http://www.inno-chem.com.cn/search.php?searchkey=+U141075-500g.

[3] http://www.inno-chem.com.cn/search.php?searchkey=P104467-500g.

[4] Y. Zheng, Z. Yu, H. Ou, A. M. Asiri, Y. Chen, X. Wang, Adv. Funct. Mater. **2018**, 28, 1705407.

[5] M. Zhu, S. Kim, L. Mao, M. Fujitsuka, J. Zhang, X. Wang, T. Majima, J. Am. Chem. Soc. **2017**, 139, 13234.

[6] J. Ran, W. Guo, H. Wang, B. Zhu, J. Yu, S. Z. Qiao, Adv. Mater. **2018**, 30, 1800128.

[7] L. Kong, Y. Ji, Z. Dang, J. Yan, P. Li, Y. Li, S. F. Liu, Adv. Funct. Mater. **2018**, 28, 1800668.

[8] W. Lei, Y. Mi, R. Feng, P. Liu, S. Hu, J. Yu, X. Liu, J. A. Rodriguez, J.-o. Wang, L. Zheng, K. Tang, S. Zhu, G. Liu, M. Liu, Nano Energy **2018**, 50, 552.

[9] Z. Shen, S. Sun, W. Wang, J. Liu, Z. Liu, J. C. Yu, J. Mater. Chem. A **2015**, 3, 3285.

[10] Z. He, C. Sun, S. Yang, Y. Ding, H. He, Z. Wang, J. Hazard. Mater. **2009**, 162, 1477.
